# Performance of soccer players under acute physical fatigue: An approach based on cognitive, tactical and physical aspects

**DOI:** 10.1016/j.heliyon.2024.e30516

**Published:** 2024-04-30

**Authors:** Israel Teoldo, Felipe Dambroz, João Brito

**Affiliations:** a Centre of Research and Studies in Soccer (NUPEF), Universidade Federal de Viçosa, Department of Physical Education, Viçosa, Brazil; b Portuguese Football Federation, Portugal Football School, Oeiras, Portugal

**Keywords:** Effort, Perceived exertion, Performance, Decision-making, Skill, Football

## Abstract

This study aimed to verify whether peripheral perception, tactical behaviour, and physical performance are influenced by acute physical fatigue in soccer players. The study included 24 trained soccer players (18.6 ± 1.5 years) from two Brazilian clubs. The TSAFT90 test was used to induce acute physical fatigue. The results showed that physical fatigue did not affect peripheral perception (p = 0.360). Regarding tactical behaviour, improved efficiency was observed for the principles of offensive coverage (p = 0.029), width and length with the ball (p = 0.044), and concentration (p = 0.008). On the other hand, a reduction was observed in the number of tactical actions of offensive coverage (p = 0.020) and recovery balance (p = 0.042). Also, improved accuracy in the principles of defensive balance (p = 0.009), recovery balance (p = 0.021) and defensive unity (p = 0.003) occurred under physical fatigue. A reduction in the physical performance outcomes total distance covered (p < 0.001), average speed (p < 0.001), sprints (p = 0.029), number of accelerations (p = 0.008) and decelerations (p = 0.008) were also detected. The internal (p < 0.01) and external (p < 0.01) workload was higher under physical fatigue. Overall, acute physical fatigue did not influence peripheral perception. However, physical performance was reduced under fatigue, the perceived effort increased, and tactical behaviours were affected by decreasing tactical actions performed near the ball, increasing errors in defensive movements in the lateral corridors and the last defensive line, and improving offensive tactical actions performance.

## 1 Introduction

Soccer is characterised by the concurrent cooperation and opposition between players and teams across the field [1]. During a match, players face situations in which they need to select and take decisions within several contextual alternatives and according to the collective tactical and strategic purposes of the team [2,3]. Therefore, players are required to perform efficient tactical behaviours to identify appropriate solutions to the challenges that are systematically presented by the game itself, particularly regarding space occupation [4].

In order to develop efficient tactical behaviours and achieve high levels of performance, players should be capable of detecting the information available in the environment, anticipating actions, making assertive decisions, and positioning and moving adequately across the playing field [5]. In this regard, soccer players who possess well-developed perceptual-cognitive skills may decide better when facing the problems posed by the game [6,7].

Among these skills, peripheral perception is considered essential, because it contributes to performing more efficient actions by the players [8,9]. According to Friedenberg and Silverman [10], peripheral perception refers to the cognitive mechanism by which individuals collect and interpret environmental information within the visual field. This metric is quantified in degrees and relates to the capacity to detect stimuli in the left and right visual fields while simultaneously focusing central vision on a specific point.

In soccer, peripheral perception enables the player to detect information about teammates and opponents positioned in the lateral corridors, either near or distant from the ball [4]. In general, studies addressing peripheral perception in soccer emphasised that players with greater peripheral perception display superior tactical behaviours [9,11]. Also, the amount of information drawn throughout the visual field influences players’ ability to move at higher intensities [12]. These findings suggest the importance of superior peripheral perception abilities in soccer.

The complexity and dynamics of the game generate a high amount of information and demands; though, players have to take successive decisions that need to be processed and taken under limited time- and space-constraints, besides the execution of constant technical and physical actions at high intensities in short periods of time [13]. Therefore, soccer players are exposed to periods of cognitive and physical overloads during training sessions and match-play, which may influence individual and collective performance [14,15]. Indeed, Coutinho and colleagues (2018) showed that players employed more synchronized tactical behaviours during small-sided games (5 vs. 5) played under muscle fatigue, meaning that players moved closer to each other to reduce the need of high-intensity actions. These findings were justified by the adoption of a collective strategy aiming to mitigate the negative effects of muscle fatigue, leading players to positioning themselves across the field by providing more security in defensive actions. Thus, when experiencing physical fatigue, players may be inclined to choose strategies that demand less physical effort, even if such behaviour might not be the most appropriate tactical and technical option for that specific moment of the game [16,17].

During a match, acute physical fatigue manifests through substrate depletion, dehydration, hyperthermia, electrolytes, and disturbances in acid-base balance, which are often associated with decrements in performance [18]. Additionally, there is evidence suggesting that acute physical fatigue induced by intense exercise may result in decrements in cognitive processes (indicative of supraspinal effect) [19]. For instance, Alder and colleagues (2021) showed that physical load can reduce anticipatory accuracy and alter visual perception in skilled soccer players. Similarly, Klatt and Smeeton (2021) demonstrated that the intensity of exercise on a cycle ergometer can influence soccer players' capacity to recognize detailed information in the peripheral field, although no differences in decision-making were observed. On the other hand, Teoldo and colleagues (2024) demonstrated, through laboratory tests, that peripheral vision and decision-making are not significantly influenced by physical fatigue caused by the physical demands of the game. Hakim et al. [20] showed that in healthy adults, regardless of the mental condition of the participants, an increase in perceived exertion may have a detrimental effect on subsequent endurance performance. Therefore, gaining an understanding of how acute physical fatigue affects soccer players’ movement management in the sports environment could offer valuable insights for researchers, coaches, and practitioners, aiding in the adoption of more “conservative” strategies to maintain performance.

Despite the relevance of this topic and the increasing interest in understanding the impacts of mental and physical fatigue in soccer [21,22], there remains a necessity for research on how cognitive and tactical aspects are influenced by physical fatigue, particularly concerning the analysis of players’ decisions and behaviours, as a result of the interaction between several factors, ideas and knowledges [23,24]. Therefore, the present study aimed to investigate whether and how peripheral perception, tactical behaviour, and physical performance are influenced by acute physical fatigue in soccer players. Accordingly, our hypothesis was that soccer players would exhibit decreased peripheral perception, and diminished tactical behaviour and physical performance following a protocol that induces physical fatigue. The hypothesis presented above is based on studies that indicate that athletes reduce high-intensity actions under acute physical fatigue [25] and adopt strategies that demand less physical effort [16,17].

## 2 Methods

### 2.1 Sample

The sample consisted of 24 male soccer players with training experience (mean age: 18.3 ± 1.5 years) from two Brazilian clubs [26]. To be included, players needed to be actively engaged in structured training, attending a minimum of three 90-min sessions per week. The software G*Power 3.1.9.4® was utilized to determine a minimum sample size of 31 participants, based on a power (1- β) of 0.85, alfa (α) of 0.05, and effect size (ES) of 0.5 (moderate), as outlined in the scientific literature [27]. The effect size chosen was determined by referencing a published study that employed a comparable design to investigate the effects of physical fatigue on cognitive performance of youth soccer players [28]. The actual sample size used in this study was smaller than the estimated one, primarily due to data collection occurring at the end of 2019, just before the SARS-CoV-2 pandemic. The pandemic situation required a temporary halt in research activities and subsequently led to a reduction in sample size.

### 2.2 Ethical procedures

Participants were briefed on the study's purpose in advance. This study received approval from the Ethics Committee in Research with Human Subjects (No. 3.208.190) and adhered to the guidelines of the National Health Council (CNS 466/2012) and the Declaration of Helsinki (2013). Participants aged 18 years and older provided written informed consent to participate, while minors signed an assent form and their legal guardians signed an informed consent form.

### 2.3 Instruments and procedures

#### 2.3.1 Experimental design

**Fig. 1:**
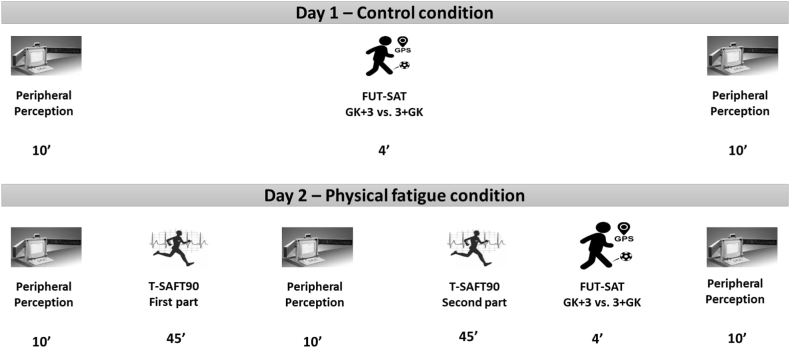
The representation of experimental design.

Participants were evaluated on two separate days (Fig. 1).

During the first session, referred to as the “Control” condition, all participants underwent a series of assessment in a predetermined order: i) evaluation of peripheral perception; ii) completion of the FUT-SAT field test (GK+3 vs. 3+GK); and iii) another peripheral perception test. In the field test, each team comprised one defender, one midfielder, and one forward. Player selection and team formations were determined by the coach based on the players' tactical, technical, and physical abilities.

The second day, named as the “Physical Fatigue” condition, took place 21 days subsequent the initial visit to minimize the influence of learning and working memory on the assessment [29]. All participants completed a series of tests in the following sequence: i) peripheral perception test; ii) SAFT^90^ test – first part; iii) peripheral perception test; iv) T-SAFT^90^ test – second part; v) FUT-SAT field test (GK+3 vs. 3+GK); and vi) peripheral perception test. The intervals between the tests lasted roughly 1 min, reflecting the average time participants required to move between testing locations. The rate of perceive exertion (RPE) was collected 30 min after the first and second days of sampling. Participants were advised to abstain from participating in physical exercise and consuming caffeinated or alcoholic beverages within the 48-h period preceding the interventions. Also, they were instructed to ensure they had 6–8 h of sleep on the night before. During the evaluation week, the players' training routine was altered so they did not engage in training sessions or participate in competitive matches over the weekend. To standardize participants' food intake, a uniform meal was provided 60 min before the tests commenced to fulfil approximately 18 % of each participant's energy requirements (approximately 380 kcal, 68 g of carbohydrates, 11 g of proteins, and 7 g of fat). Participants were allowed to drink water *ad libitum* during the experiment. The average ambient temperature and air humidity during the physical fatigue protocol days were 21 ± 1 °C and 57 ± 1 %, respectively.

#### 2.3.2 Physical fatigue induction protocol

The T-SAFT^90^ test was employed as a protocol to induce physical fatigue. This test emulates the physical demands of a 90-min competitive soccer match, aiming to reproduce the metabolic requirements of the game and elicit comparable internal and external match load responses. This task was selected for its capacity to induce muscle damage through actions like accelerations, decelerations, and changes of direction (1269 changes in speed, 888 changes in direction (180°), 444 cutting manoeuvres, and 12 jumps), ultimately leading to muscular fatigue as a result. The T-SAFT90 includes ball-related activities such as running, dribbling, shooting, passing, and jumps (including 0.36 km of ball dribbling, 24 short passes, and 12 shots on target) performed intermittently at different speeds to simulate a real soccer match [30].

The fatigue protocol involved two 45-min exercise sessions separated by a 15-min passive rest period during halftime. Each half were segmented into six blocks, each lasting 15 min.

#### 2.3.3 Peripheral perception

The assessment of participants' peripheral perception was conducted using the Vienna Test System's peripheral perception test (PP) – version S1 [31]. The test is displayed on two peripheral panels attached to the left and the right sides of the computer screen. These panels illuminate green light diodes, signaling participants to promptly react by pressing a pedal with their dominant foot when a complete vertical light line appears. At the same time, participants engage in a central tracking task, which involves tracking a moving ball through a crosshair on the screen (participants are directed to focus their attention on the center of the visual field). The crucial peripheral stimulus is presented multiple times and in various locations throughout the evaluation.

The primary metric employed in this assessment was the visual field, derived from the addition of the right and left visual angles. The evaluation of the visual field considered factors such as the locations of the visual stimulus and target, as well as the distance between the participant's head and the equipment. Additionally, other variables such as tracking deviation (i.e., time of deviation from the object being followed on the screen) and reaction time ((i.e., time taken to respond to peripheral stimuli) were recorded. The assessment lasted for a total duration of 10 min.

#### 2.3.4 Tactical behaviour

**Fig. 2:**
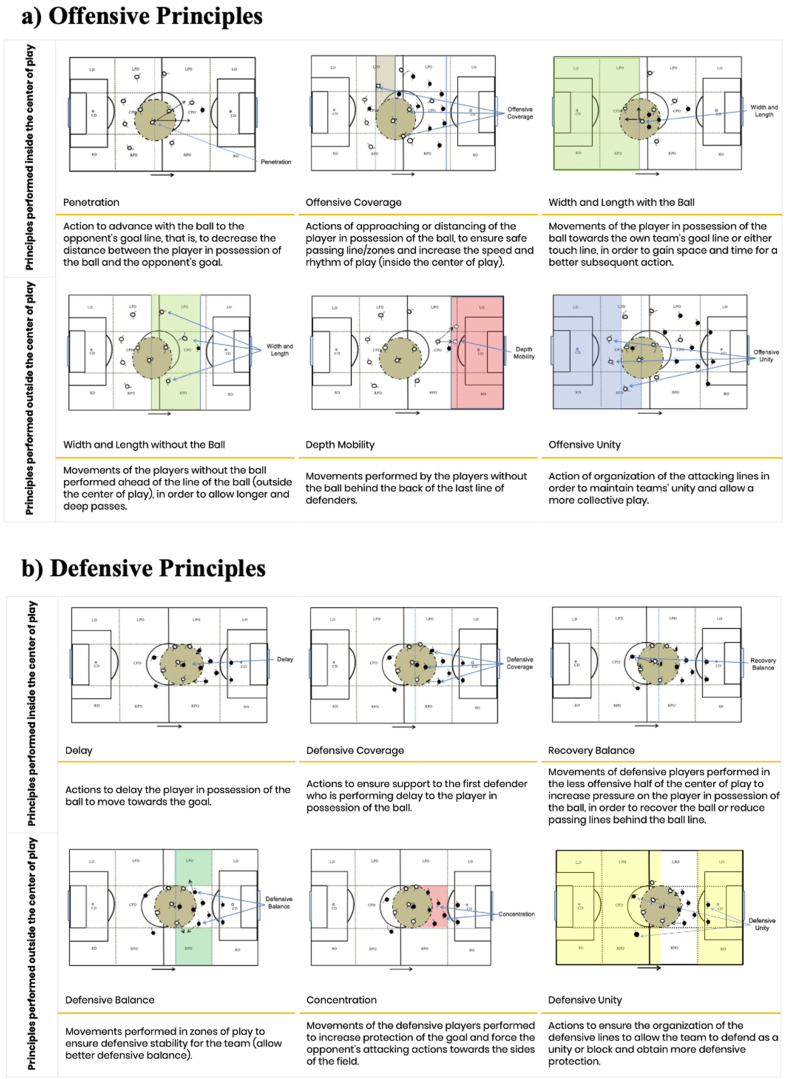
Description of the core tactical principles.

Participants' tactical behaviour was assessed using the System of Tactical Assessment in Soccer (FUT-SAT) [32]. The tactical evaluation carried out using FUT-SAT is grounded on the ten tactical principles of soccer, comprising five offensive (Fig. 2a) and five defensive principles (Fig. 2b), both with and without possession of the ball, executed within or beyond the centre of play. The centre of play is a dynamic spatial point defined by a 9.15 m radius from the ball's position, where game actions and decisions unfold rapidly [4]. FUT-SAT evaluations were conducted within a small-sided game format (GK+3 vs. 3+GK) on a 36 m length and 27 m wide pitch, lasting for 4 min and following the official soccer regulations.

The FUT-SAT comprises two Macro Categories: the Observation Macro Category and the Outcome Macro Category. The Observation Macro Category includes variables associated with Tactical Principles, the Location of Action on the Field, and the Outcome of the Action. Conversely, the Outcome Macro Category encompasses the Tactical Performance Index (TPI) as a Performance Indicator, Tactical Actions as an indicator of the number of tactical actions, Percentage of Accuracy as a measure of success and quality of tactical actions, and Place of Action Related to the Principles (PARP), totalling 52 variables. The Outcome Macro Category enables the assessment of soccer players' tactical behaviour by examining their tactical actions with and without the ball, aligning with the core tactical principles of soccer. This category is named as such because its variables rely on data derived from the Observation Macro Category.

The FUT-SAT protocol involves three main procedures. Initially, player actions during a game are analysed, with ball possession used as the basis to distinguishing between defensive and offensive phases. The second step entails evaluating, categorizing, and documenting tactical actions based on spatial references on the field. The final step involves calculating variables within the “Tactical Actions” and “Percentage of Correct Actions” categories. In this study, the effectiveness and frequency of tactical actions were assessed using FUT-SAT variables such as percentage of accuracy and number of tactical actions. Subsequent to data collection, analysis was conducted following the methodology outlined by Costa and colleagues (2011).

#### 2.3.5 Workload

Internal load: In the present study, the adapted CR10 scale introduced by Foster et al. [33] was employed to assess the session's rating of perceived exertion (sRPE), with reference points ranging from 0 (rest) to 10 (maximum effort). The RPE scale was utilized to evaluate participants' perceived exertion levels during exercise. Prior to the study's beginning, all athletes were introduced to and became familiar with this scale. At the conclusion of the first and second days of data collection (30 min after the tests), players were asked to answer the question “How was your workload?”. The participants verbally informed the RPE score to the researcher in charge. Arbitrary units (AU) were determined by multiplying the modified Borg's CR10 scale by the overall duration of the session.

External load: The session's external load was assessed based on the distance covered (m), and the number of accelerations (AU) and decelerations (AU). GPS units (SPI-HPU, GPSports®, Canberra, Australia) with a sampling rate of 5 Hz for player position tracking on the field and 100 Hz for the accelerometer were used in this study. These units were linked to a triaxial accelerometer, allowing for precise data collection through satellite signals and positional data.

Each player wore a GPS unit attached to their upper back using an elastic harness. The GPS units were activated 15 min prior to the warm-up following the manufacturer's guidelines. To maintain consistency, players use the same device throughout the session to minimize inter-unit discrepancies. Previous studies had already established the validity and accuracy of these devices [34].

#### 2.3.6 Physical performance

During the field test, players wore GPS units (SPI-HPU, GPSports®, Canberra, Australia) to collect physical performance data, including distance covered, average speed, sprints, number of accelerations, and number of decelerations*.* In total, 24 units from a global tracking system were utilized for data collection. Intensity zones were classified according to the research conducted by Bradley et al. [35], with running speeds segmented as follows: Standing (<0.7 km/h^−1^), Walking (0.8–7.1 km/h^−1^), Jogging (7.2–14.3 km/h^−1^), Running (14.4–19.7 km/h^−1^), High-Speed Running (19.8–21.1 km/h^−1^), and Sprinting (>25.1 km/h^−1^). To ensure reliability, players wore the same unit in both scenarios. The GPSports® (SPI-IQ) software was used for processing and analyzing physical performance data.

### 2.4 Statistical analysis

Descriptive analysis, including means and standard deviations, was conducted on the sample. The normality of data distribution for all variables was assessed using the Shapiro-Wilk test. To compare variables (tactical behaviour, physical performance, and RPE) between the control and physical fatigue conditions, the paired *t*-test and Wilcoxon test were utilized. For the peripheral perception variables in the control condition, the paired *t*-test and Wilcoxon test were employed, while a repeated measures ANOVA was used to assess the peripheral perception variables in the physical fatigue condition. The effect size was calculated using generalized eta squared (η2) [36]. Confidence intervals (CI) of 95 % were calculated to quantify the magnitude of mean difference, and the effect sizes were obtained using Cohen's d. The magnitudes of the effect sizes were classified as: null (<0.20), small (0.21–0.60), medium (0.61–1.20), and large (>1.20) [37].

Test-retest reliability was conducted with a 21-day interval for reanalysis to reduce potential biases related to task familiarity [29]. Cohen's Kappa was employed for reliability calculation. The analysis involved the re-evaluation of 385 tactical actions, which represented 11 % of the total sample, exceeding the minimum threshold recommended in the literature [38]. Intra- and inter-observer reliability ranged between 0.84 and 0.95, and 0.83 and 0.98, respectively. These values are classified as “Almost Perfect” (0.81–1.00) by literature [39].

A significance level of p < 0.05 was set for all statistical analyses. IBM SPSS (Statistical Package for Social Sciences) for Windows®, version 24.0, was employed for all statistical procedures.

## 3 Results

### 3.1 Peripheral perception

There were no significant differences in peripheral perception variables observed across the “control” and “physical fatigue” conditions (Table 1).

**Table 1:** Comparison between the results pre and post the peripheral perception test in the control condition, and comparison among the results pre, interval, and post the peripheral perception test in the physical fatigue condition.

Peripheral Perception	Control								Physical Fatigue										
	Pre			Post			p	d	Pre			Half-time			Post			p	η2
	M		DP	M		DP			M		DP	M		DP	M		DP		
Visual Field (°)	184,39	±	6,17	185,24	±	8,93	0,556	0.11	182,75	±	7,96	184,27	±	8,23	184,49	±	9,390	0,360	0,02
Tracking Deviation (pixels)	5,58	±	1,30	5,59	±	1,38	0,568	0.01	6,36	±	2,73	5,63	±	1,36	6,31	±	2,290	0,595	0,01
Reaction Time (s)	0,59	±	0,07	0,58	±	0,08	0,636	0.13	0,59	±	0,07	0,58	±	0,07	0,57	±	0,07	0,663	0,01

### 3.2 Tactical behaviour

The participants performed a lower number of tactical actions of the principles of offensive coverage (*t*_(*23*)_ = 2.51; p = 0.020; medium effect), recovery balance (*t*_(*23*)_ = 2.15; p = 0.042; small effect), as well as overall offensive (*z* = −2.62; p = 0.009; medium effect) and defensive actions (*z* = −2.58; p = 0.010; medium effect) in the “physical fatigue” condition (Table 2).

**Table 2:** Comparison of the frequency of tactical principles between the control and physical fatigue conditions.

Tactical principles	Control			Physical Fatigue			CI 95 %	p	d
	M		SD	M		SD	Lower - Upper		
**Offensive**									
Penetration	4.9	±	3.3	4.0	±	2.2	−0.3 to 2.1	0.134	0.33
Offensive Coverage	9.0	±	3.8	6.4	±	4.2	0.5 to 4.8	0.020^a^	0.65
Depth Mobility	1.8	±	2.1	1.2	±	1.6	−1.0 to 0.5	0.289	0.31
Width and Length with the Ball	1.5	±	1.3	1.8	±	1.4	−0.5 to 1.0	0.466	0.22
Width and Length without the Ball	11.9	±	5.8	10.3	±	4.7	−1.0 to 4.3	0.218	0.31
Offensive Unity	7.2	±	4.3	6.6	±	3.5	−1.2 to 2.4	0.506	0.15
**Defensive**									
Delay	7.5	±	4.4	6.4	±	3.6	−0.9 to 3.1	0.279	0.27
Defensive Coverage	2.7	±	1.7	2.2	±	1.9	−1.5 to 0.5	0.247	0.26
Defensive Balance	4.3	±	3.0	3.6	±	3.3	−2.0 to 0.0	0.156	0.21
Recovery Balance	3.1	±	2.2	2.0	±	1.4	0.0 to 2.1	0.042^a^	0.58
Concentration	5.8	±	3.2	4.5	±	2.7	−3.0 to 0.5	0.134	0.42
Defensive Unity	16.4	±	3.9	14.5	±	4.8	−0.4 to 4.2	0.094	0.44
**Totals**									
Offensive	36.2	±	9.4	30.1	±	9.1	−10.5 to −2.5	0.009^a^	0.65
Defensive	39.6	±	10.4	33.1	±	10.0	−11.5 to −2.5	0.010^a^	0.63

a Significant difference at p < 0.05.

In the “physical fatigue” condition, higher values of tactical behaviour efficiency were found for the principles of offensive coverage (*z* = −2.18; p = 0.029; small effect), width and length with the ball (*z* = −2.01; p = 0.044; medium effect) and concentration (*z* = −2.65; p = 0.008; large effect). Conversely, reduced efficiency was noted for the tactical principles of defensive balance (*z* = −2.62; p = 0.009; small effect), recovery balance (*z* = −2.32; p = 0.021; medium effect) and defensive unity (*z* = −2.98; p = 0.003; medium effect), as well as for the overall defensive tactical efficiency (*t*_(*23*)_ = 4.13; p < 0.001; medium effect) (Table 3).

**Table 3:** Comparison of the of percentage accuracy of tactical principles between the control and physical fatigue conditions.

Tactical Principles	Control			Physical Fatigue			CI 95 %	p	d
	M		SD	M		SD	Lower - Upper		
**Offensive**									
Penetration	89.4	±	12.1	80.7	±	27.3	−14.2 to 9.1	0.629	0.41
Offensive Coverage	92.9	±	11.7	98.2	±	4.7	0.0 to 10.6	0.029^a^	0.59
Depth Mobility	91.0	±	19.9	69.3	±	39.8	−100.0 to 6.7	0.115	0.69
Width and Length with the Ball	57.3	±	47.9	93.3	±	23.2	0.0 to 75.0	0.044^a^	0.96
Width and Length without the Ball	88.7	±	11.3	86.5	±	14.4	−9.7 to 5.5	0.651	0.17
Offensive Unity	91.5	±	11.3	77.0	±	22.8	−19.1 to 0.0	0.064	0.81
**Defensive**									
Delay	69.6	±	19.8	73.6	±	26.9	−25.0 to 11.7	0.493	0.17
Defensive Coverage	89.6	±	18.0	83.3	±	30.9	−20.0 to 4.2	0.539	0.25
Defensive Balance	79.9	±	22.7	69.4	±	28.6	−48.5 to −4.3	0.009^a^	0.40
Recovery Balance	64.8	±	36.4	24.6	±	40.0	−81.7 to 27.8	0.021^a^	1.05
Concentration	58.9	±	30.3	94.4	±	13.4	6.3 to 41.7	0.008^a^	1.52
Defensive Unity	76.5	±	17.9	59.4	±	21.4	6.9 to 27.4	0.002^a^	0.87
**Totals**									
Offensive	87.9	±	9.0	85.2	±	7.8	−1.2 to 6.8	0.162	0.33
Defensive	76.3	±	11.9	63.6	±	14.5	6.1 to 18.5	<0.001	0.96

a Significant difference at p < 0.05.

### 3.3 Workload

Internal load: Participants displayed higher sRPE during the physical fatigue condition (673.66 ± 23.10) than in the control condition (4.0 ± 0.87) (z = −4.06 p < 0.001; large effect).

External load: In the “physical fatigue” condition, the participants displayed higher distance covered (10852.82 ± 12.76), number of accelerations (792.00 ± 1.92) and number of decelerations (578.88 ± 1.45) than in the control condition (500.68 ± 7.13) (*t*_(*23*)_ = 952.10; p < 0.001; large effect), (47.13 ± 1.08) (*t*_(*23*)_ = 425.38; p < 0.001; large effect) and (33.50 ± 0.93) (*t*_(*23*)_ = 440.11; p < 0.001; large effect), respectively.

### 3.4 Physical performance

Under physical fatigue, participants covered less overall distance (*t*_(*23*)_ = 5.95; p < 0.001; large effect), as well as distances jogging (*t*_(*23*)_ = 3.80; p = 0.001; medium effect), running (*t*_(*23*)_ = 4.11; p < 0.001; large effect), high-speed running (*z*_(*23*)_ = −2.23; p = 0.026; small effect), sprint (*z*_(*23*)_ = −2.03; p = 0.043; small effect). In addition, participants displayed a drop in average speed (*t*_(*23*)_ = 6.01; p < 0.001; large effect), peak speed (*t*_(*23*)_ = 2.65; p = 0.014; medium effect) as well as in the number of sprints (*z*_(*23*)_ = −2.19; p = 0.029; medium effect), accelerations (*t*_(*23*)_ = 2.93; p = 0.008; medium effect) and decelerations (*t*_(*23*)_ = 2.93; p = 0.008; medium effect) (Table 4).

**Table 4:** Comparison of physical data between the control and physical fatigue conditions.

Physical Performance	Control			Physical Fatigue			CI 95 %	*p*	*d*
	*M*		*SD*	*M*		*SD*	*Lower – Upper*		
Total distance covered (m)	500.7	±	34.9	436.0	±	62.5	42.2 to 87.1	<0.001	1.28
Standing (m)	1.6	±	0.6	1.5	±	0.6	−0.2 to 0.5	0.473	0.21
Walking (m)	179.3	±	17.1	190.3	±	30.5	−3.0 to 21.6	0.137	0.45
Jogging (m)	235.2	±	28.9	190.3	±	67.0	20.5 to 69.4	0.001^a^	0.87
Running (m)	70.4	±	20.8	46.8	±	17.2	11.8 to 35.6	<0.001	1.24
High-speed running (m)	13.7	±	11.5	7.2	±	10.5	−13.5 to −0.7	0.026^a^	0.59
Sprinting (m)	0.6	±	1.4	0.1	±	0.5	−0.7 to 0.0	0.043^a^	0.42
Average Speed (km/h)	7.4	±	0.5	6.4	±	0.9	0.6 to 1.3	<0.001	1.28
Peak speed (km/h)	22.2	±	2.3	20.7	±	1.8	0.3 to 2.7	0.014^a^	0.73
Sprints (AU)	1.1	±	1.1	0.5	±	0.2	−1.5 to 0.0	0.029^a^	0.74
N. of Accel. (AU)	47.1	±	5.3	42.0	±	9.4	1.5 to 8.8	0.008^a^	0.67
N. of Decel. (AU)	33.5	±	4.6	29.9	±	7.1	1.1 to 6.2	0.008^a^	0.61

a Significant difference at p < 0.05.

## 4 Discussion

The aim of the present study was examining the potential impact of acute physical fatigue on the peripheral perception, tactical behaviour and physical performance of soccer players. Our findings indicated that peripheral perception was not affected by acute physical fatigue. However, acute physical fatigue changed players’ tactical behaviour, namely a lower number of tactical actions performed inside the centre of play and an increase in tactical behaviour efficiency displayed for the principles of width and length with the ball, offensive coverage, and concentration. In addition, under acute physical fatigue, the players made more mistakes in defensive tactical actions performed in the lateral corridors of the last defensive line. Also, a decrease in collective defensive compression was observed. Lastly, under fatigue, players covered less distance and reduced the intensity of their movements and displacements but displayed higher perceived effort in this condition.

With respect to the peripheral perception skills, no changes were observed in any of the parameters assessed between control and fatigue conditions. This finding might be justified by the players' ability to retain task engagement throughout the entire peripheral perception assessment protocol. Therefore, it is possible to infer that tasks involving attentional focus are not affected by physical fatigue but vary as a function of individual expertise and cognitive effort [22,40]. Nevertheless, it is noteworthy that players were exposed to stimuli for only 10 min [31]. Therefore, taking into account that during a competitive match players may remain vigilant for longer periods as they adjust strategic and tactical behaviours continuously in response to contextual changes [6], increasing the time of peripheral perception assessment might have an effect on players’ response patterns.

Also, in relation to peripheral perception, Lemmink and colleagues (2005) identified a positive correlation between peripheral perception and running capacity in soccer. However, in the present study, peripheral perception remained unchanged even in situations in which players were physically exhausted and decreased the intensity of movements and displacements. Thus, peripheral perception might be less related to physical performance dynamic situations, such as small-sided games, possibly because this context can provide players with greater freedom to adjust their efforts and change the playing time [21,16].

Based on these findings, changes in peripheral perception are more likely related to a high cognitive demand rather than with the physical demands of the task. This assumption is supported by studies that suggested that mental load and task complexity reduce the individuals’ ability to capture peripheral information [41]. More recently, the study by Kunrath and colleagues (2020) showed that college soccer players induced to mental fatigue displayed a reduction in the visual field, which resulted in loss of tactical behaviour efficiency and increased distance covered in small-sided and conditioned games. Hence, high cognitive demands seem to affect peripheral perception mechanisms [4,14], something that physical fatigue, as induced by the T-SAFT^90^, was not able to promote.

In the current study, we included internal and external workload variables that are considered sensitive in detecting fatigue [42]. We observed a higher mean of sRPE, distance, number of acceleration and deceleration under physical fatigue than in the control condition. Several studies on amateur and professional soccer players have found abrupt alterations in muscle cells after high values of workload [18] as well as decrements in the amount of high-intensity running, distance, sprint ability and capacities of acceleration and deceleration [43,44]. Our results also found a significant decline in physical performance during the field test of FUT-SAT. These findings support that the participants face scenarios of acute physical fatigue after being subjected to this physical fatigue protocol.

Our findings also showed that acute physical fatigue induced the players to cover less distances and to decrease the intensity of displacements, which consequently led to a lower number of tactical actions. In turn, the decrease in the number of tactical actions essentially occurred in behaviours performed inside the centre of play, in which the game is played at a higher time, and where decisions and movements must occur faster [4]. Accordingly, although players are capable to maintain collective tactical organization when physically fatigued [21], our findings suggest that acute physical fatigue affected players’ individual tactical behaviour, especially in tactical actions performed under limited space and time.

As for the improvement in the performance of the tactical principle of concentration, the results indicated that induced physical fatigue resulted in greater efficiency to protect the goal. This improvement may be interpreted as an attempt to ensure collective defensive stability, decrease the likelihood of conceding goals and reduce the need to perform movements throughout the playing field [13]. Similar results were found by Barte and colleagues (2020), who submitted players to a fatigue-inducing protocol, and observed that players adopted a more passive marking guarding approach and were less motivated to take risks by intercepting passes when physically fatigued. Correspondingly, when physically exhausted, players seek to ensure defensive stability, backing off the defensive block and waiting until the opponent with the ball takes the initiative.

During the defensive phase, players are dependent on the opponents' decisions to react [45] though, the adoption of a more passive guarding approach may require longer reaction time and result in greater demands of intensity in actions, as compensation. Yet, the state of physical fatigue may impair performance of high-intensity actions [46], which conforms to the efficiency drop observed for the defensive tactical principles, especially for the principles of defensive balance, recovery balance and defensive unity, which take place in zones of the field where the space players' have to manage is greater, and that demand more abstract decisions. Thus, acute physical fatigue may have led players to make more mistakes and to display greater difficulty to perform movements in the lateral corridors and in the last defensive line, which may be related to the fact that the performance of core defensive tactical principles demands constant movements by the players across the playing space, in order to regain possession and reducing the opponents’ action possibilities [4].

With respect to the improvement in the performance of the offensive tactical principles of width and length with the ball and offensive coverage, players under physical fatigue are advised to adapt their tactical and technical actions to increase safety when performing offensive actions. Creating passing lines and carrying the ball towards the side lines or their own goal are actions whose purpose is to buy time and space [47]. Consequently, it is possible that players may have refined these offensive tactical actions due to the decrease in physical performance, to ensure the progression of the attacking sequence, reducing the risk to lose ball possession and avoiding pressure from the opposing team [48]. As a result, and in addition to the improved compression by the opposition, players in possession of the ball who improved their actions of width and length with the ball and offensive coverage, seem to have used spaces in the field poorly occupied by the opposing team, and thus gained more space and were less pressed when attempting to build up attacking plays [16,49].

The results of this study provide important contributions for the understanding of the tactical, physical, and cognitive aspects of soccer players under physical fatigue. These results can be improved and better understood by using experimental designs that include games between “control” teams (not physically fatigued), as well as between physically fatigued teams. Regarding limitations, the present study did not incorporate counterbalancing of conditions, with the control condition conducted first, potentially causing an order effect. However, it is important to note that the observed variations between the experimental conditions cannot be solely attributed to this particular limitation. For future research on this topic, it is also suggested the randomization of data collection days (control and experimental) to improve the interpretation of the independent and dependent variables regarding their influence on performance in the field. Thus, the independent and dependent variables could be better explored with respect to their actual influence on performance. Also, the configuration and size of the sample in the present study could be considered a limitation. Further studies may benefit from larger sample sizes and more diverse range of team configuration.

In practical terms, the results of the present study indicate that coaches should take physical exhaustion into account. Although no influence of acute physical fatigue on peripheral perception was observed, it is important to mention that participants were only assessed for 10 min. Therefore, future studies should increase the time of peripheral perception assessment. In addition, decreases were observed in players’ physical performance and defensive tactical behaviour, especially for the principles performed in zones of the field in which there is an increased demand to manage larger spaces (i.e., lateral corridors and last defensive line). Consequently, in situations in which players are physically exhausted, coaches are advised to seek for strategies to encourage the team to adopt a more purposeful game, with emphasis on retaining possession, with the intention to reduce the likelihood of defensive errors and, as a result, increase the chances of success in attacking sequences.

## 5 Conclusions

In conclusion, induced physical fatigue changed the tactical behaviour of soccer players in small-sided games, by affecting the movement dynamics and reducing the number of tactical actions performed near the ball. In addition, physical fatigue led to a drop in physical performance. However, it did not influence the ability to detect visual stimuli from the peripheral visual field, and thus did not inhibit players to capture the information that allow players to deal with the contextual variables of the game. Thus, it is possible to conclude that induced acute physical fatigue prompted tactical behaviour changes but did not affect peripheral perception of soccer players.

## 6 Practical applications

1. Acute physical fatigue does not influence the cognitive aspect of young soccer players, such as the ability to detect visual stimuli from the peripheral visual field, which did not prevent players to capture the information required for dealing with the contextual variables of the game.
2. In situations in which players are physically fatigued, the managing of larger spaces was found more challenging (i.e., lateral corridors and last defensive line).
3. Acute physical fatigue changed the tactical behaviour of soccer players in small-sided games by affecting the movement dynamics and reducing the number of tactical actions performed near the ball.
4. Given the performance implications of acute physical fatigue outlined in this study, coaches, researchers, and players may want to consider implementing more “conservative” strategies during training sessions and games to maintain performance levels.

## Funding

This study received support from SEESP-MG, FAPEMIG, CNPq, Funarbe, the Dean's Office for Graduate and Research Studies, and the Centre of Life and Health Sciences at the Federal University of Viçosa, Brazil. Additionally, funding for this research was provided in part by the National Secretariat of Football and Fan Rights (SNFDT) through the Academy & Football Program, as well as by the Coordenação de Aperfeiçoamento de Pessoal de Nível Superior- Brasil (10.13039/501100002322CAPES) – Finance Code 001.

## Data availability statement

Data will be made available on request.

## CRediT authorship contribution statement

**Israel Teoldo:** Writing – review & editing, Supervision, Methodology, Conceptualization. **Felipe Dambroz:** Writing – original draft, Visualization, Investigation, Formal analysis. **João Brito:** Writing – review & editing, Visualization, Supervision.

## Declaration of competing interest

The authors declare that they have no known competing financial interests or personal relationships that could have appeared to influence the work reported in this paper.
